# Supplementary figures and images for: Associated bacteria of Botryococcus braunii (Chlorophyta)
Source: PeerJ. 2019 Mar 27;7:e6610. doi: 10.7717/peerj.6610 (PMC6441321; doi:10.7717/peerj.6610)

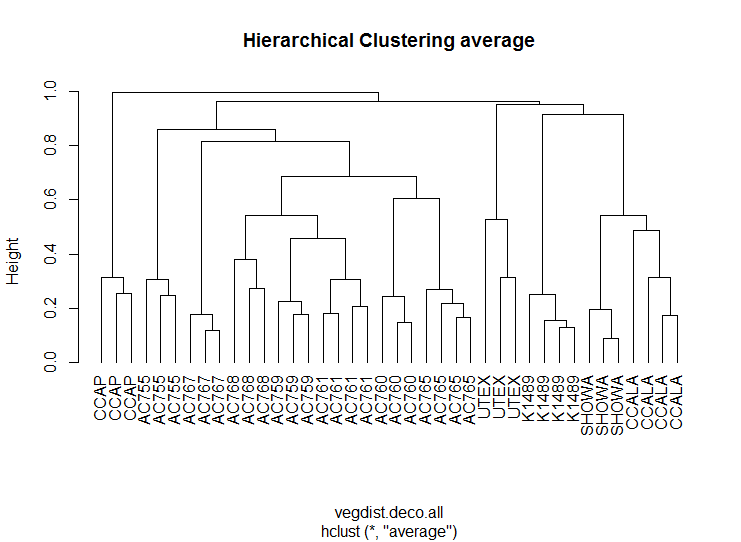

Supplement: Supplemental Information 1 — Strains from CAEN culture collection (AC prefix) cluster together in comparison to the other strains. [file peerj-07-6610-s001.png]

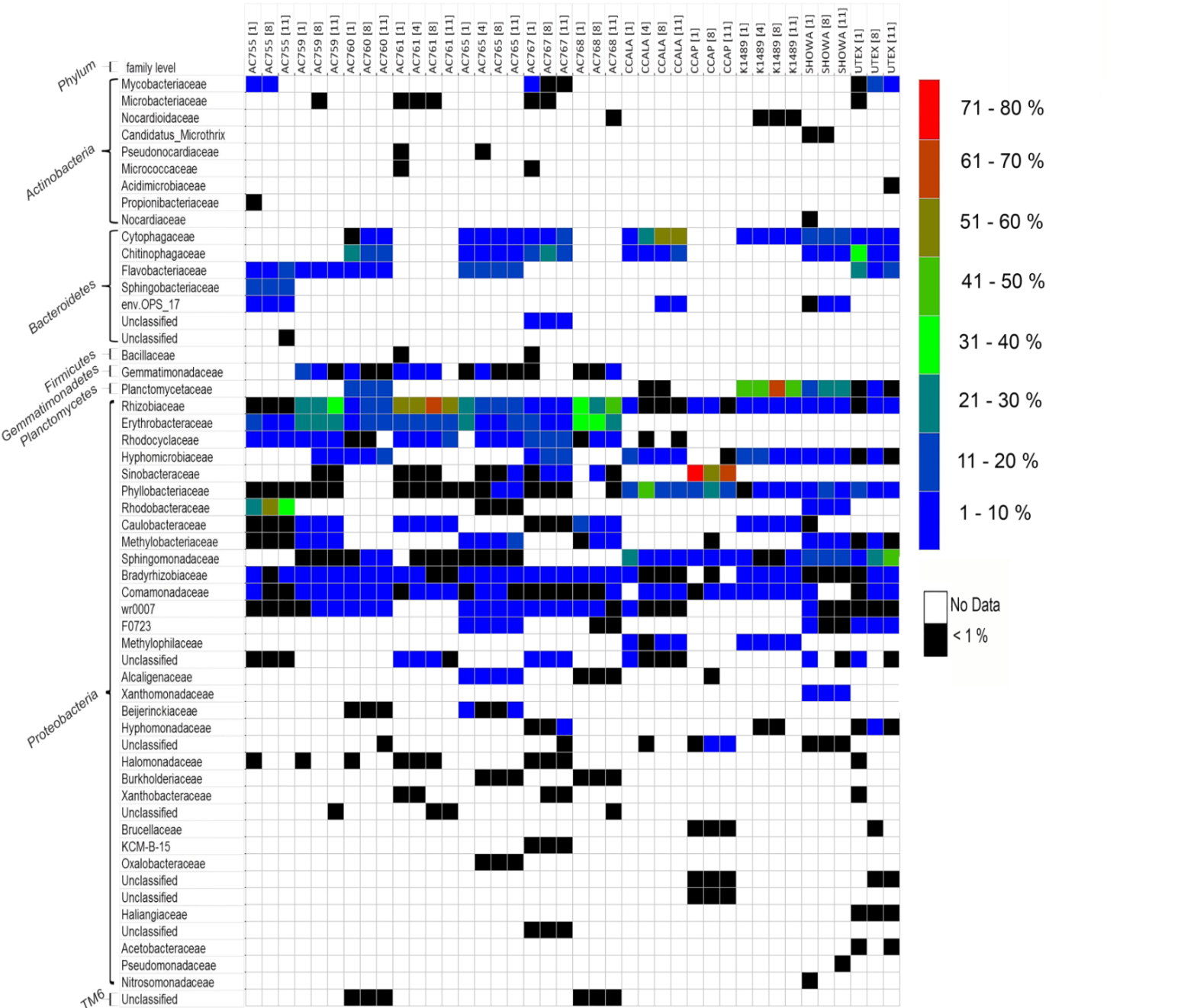

Supplement: Supplemental Information 2 — On the left, is the family taxa classification. On the right, the colour coded label describes the relative abundance in percentage. [file peerj-07-6610-s002.png]
